# Application of machine learning in CT images and X-rays of COVID-19 pneumonia

**DOI:** 10.1097/MD.0000000000026855

**Published:** 2021-09-10

**Authors:** Fengjun Zhang

**Affiliations:** College of Mechanical and Electrical Engineering, Nanjing University of Aeronautics and Astronautics, Nanjing, China.

**Keywords:** coronavirus disease 2019, computed tomography, machine learning, X-ray

## Abstract

Coronavirus disease (COVID-19) has spread worldwide. X-ray and computed tomography (CT) are 2 technologies widely used in image acquisition, segmentation, diagnosis, and evaluation. Artificial intelligence can accurately segment infected parts in X-ray and CT images, assist doctors in improving diagnosis efficiency, and facilitate the subsequent assessment of the severity of the patient infection. The medical assistant platform based on machine learning can help radiologists make clinical decisions and helper in screening, diagnosis, and treatment. By providing scientific methods for image recognition, segmentation, and evaluation, we summarized the latest developments in the application of artificial intelligence in COVID-19 lung imaging, and provided guidance and inspiration to researchers and doctors who are fighting the COVID-19 virus.

## 1 Introduction

**Figure 1:**
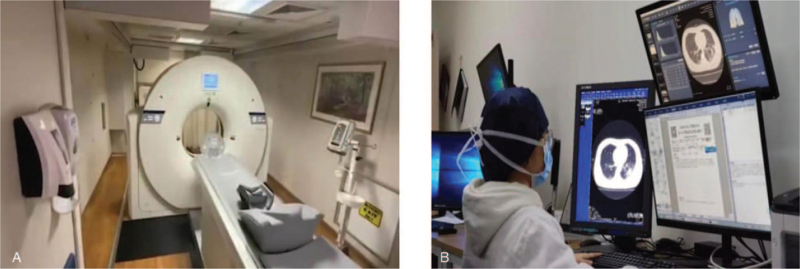
Vehicle-mounted CT machine and doctor remote operation scan. (A) Vehicle-mounted mobile CT (B) Doctors issue reports based on CT.

Coronavirus disease 2019 (COVID-19) is spreading worldwide. On March 11, 2020, the World Health Organization confirmed the worldwide pandemic of COVID-19. On July 28, 2020, the number of people infected with the new coronavirus worldwide exceeded 16.562 million, of which more than 654,000 people died, and more than 10.147 million were cured.^[1–3]^ The actual number of infections may exceed the number of confirmed cases. The reverse transcription-polymerase chain reaction (RT-PCR) test is the standard for identifying COVID-19 patients, but this process is usually time-consuming, and the initial virus concentration is not high and easy false negatives occur. Therefore, computed Tomography (CT) and X-rays also play an important role in the auxiliary diagnosis process, and they are a powerful helper in the diagnosis and judgment of disease progression.^[4]^ CT and X-ray assessment of lung infections, further testing, isolation observation, and corresponding treatment are also important.^[5]^ CT scans can assist the detection of mutated COVID-19 than RT-PCR may detect false negative and it can also be used to quantitatively assist in evaluating the treatment effect through CT scans. According to the deep learning structure and transfer learning, Lu et al^[6]^ detected pathological brains in magnetic resonance images (MRI) and introduced transfer learning to train deep neural networks. Therefore, it can be seen that machine learning is widely used in diagnosis. Fig. 1(A) shows a vehicle-mounted CT machine, which allows suspected patients to be distinguished from ordinary patients during consultation to avoid more transmission, it can also provide emergency support to areas that require medical equipment. Figure 1(B) shows the doctors performing CT scans and issuing diagnostic reports.

**Figure 2:**
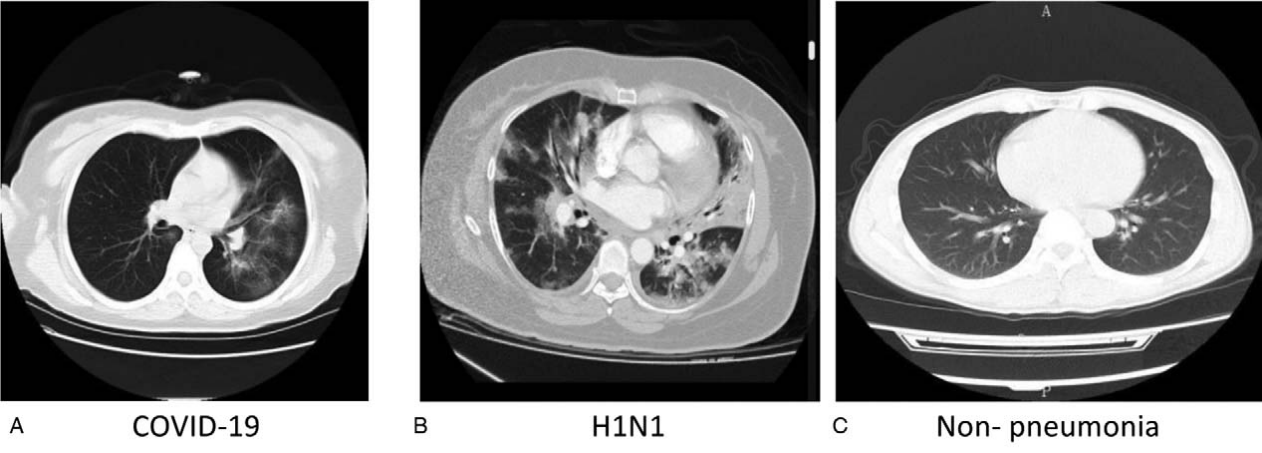
Typical cross-sectional CT image.

With the rapid development of computer technology, digital image processing technology has been widely used in the medical field, including organ segmentation and image enhancement and restoration, to provide support for subsequent medical diagnoses.^[7–9]^ Artificial intelligence establishes a neural network that simulates the human brain for analysis and learning to promote machine learning.^[10]^ It mimics the mechanism of the human brain for analysis and processing. The combination of artificial intelligence technology, deep mining of big data, and optimization of computational models has been widely used in many fields such as computer graphics, image processing, computer vision, and computer-aided design. Image visualization technology is based on the big data era, how to make full use of artificial intelligence and deep learning methods to analyze and process massive and complex medical image big data, and guide the screening, diagnosis, treatment, and image guidance of various major diseases in clinical medicine; curative effect evaluation and follow-up provide scientific methods and advanced technologies, which are major scientific problems and key technologies of cutting-edge medical imaging that urgently need to be solved in the field of medical image analysis. For example, convolutional neural networks (CNNs) and other powerful target recognition capabilities are also widely used in medical image processing. For example, it plays an active role in the diagnosis of lung nodules and the classification of benign and malignant tumors. Mature medical AI technology that can be put into clinical use is an important means to deal with the shortage of diagnosis and treatment manpower for the front line of the epidemic that is overloaded. Lung CT is extremely important for the treatment of new coronary pneumonia. Lung CT is not only an important means of diagnosing viral pneumonia, but also a powerful tool for judging the progress of the disease, changing nodes, and disease outcome. CT examination, early detection of changes in the condition, striving to intervene in time before the transition from moderate to severe, and interrupt the evolution of the disease to severe through life support treatment. For mild patients, increasing the frequency of CT examinations can also detect potential changes in the condition in time and take corresponding measures as soon as possible. Although clinical doctors cannot wait to perform a lung CT every 2 or 3 days for patients with uncertain conditions, the current clinical practice cannot meet this demand. The risks of patient transfer (many patients need to inhale oxygen at any time) and manpower requirements (new crown patients are unlikely to be pushed by their family members to perform CT, only medical staff can escort them), as well as the radiation damage of the radiological examination itself. Current clinical images: The inspection force cannot support high-frequency CT demand. Moreover, new coronary pneumonia is a “new disease” Many patients with mild symptoms have atypical lung images. Some patients with basic lung diseases (such as tuberculosis, chronic bronchitis, COPD) cover up with each other and increase diagnosis and distinction are difficult, even for senior experts. The time required for reading and diagnosis is at least 10 to 15 minutes.^[6]^ Most of these are repetitive mechanical labor. The ability to effectively identify and segment COVID-19 lung CT and distinguish other types of pneumonia is the focus of artificial intelligence applications. This can help doctors improve work efficiency, reduce high-intensity work pressure, and provide scientific data and management solutions to patients.^[11]^Table 1 shows the different lung imaging features, and Figure 2 shows the CT chest scan of COVID-19, H1H1, and no infection.

**Table 1:** Characteristics of 4 common respiratory viral infections.

Typical CT findings						
	Distribution	Consolidation	GGO	Nodule	Bronchial wall thickening	Pleural effusion
COVID-19	Periph-eral, multifocal	+++	+	Rare	Rare	Rare
RSV	Airway	+	+	Centrilobular +++	Rare	Rare
COP	Under the pleura, around the bronchus	+++	Rare	Rare	Rare	Rare
AIP	Diffuse or upper lung	Rare	+++	Rare	+++	Rare
DIP	Lower lung, periphery and under chest mold	Rare	+	Rare	Rare	Rare
Adenovirus	Multifocal	+++	+++	Centrilobular +++	Rare	Rare
H1N1	Lower lung	+++	+++	+++	Rare	+

## 2 Application of artificial intelligence in computed tomography segmentation

### 2.1 Based on lung ROI region segmentation and lung lesion segmentation

Image segmentation refers to the process of dividing an image into multiple sub-regions.^[12–17]^ The pixels in these sub-regions usually have similar attributes and do not intersect each other. Medical image segmentation is an important application field of image segmentation, and with the continuous development of modern medical technology and computer science technology, medical images are divided into special images that are different from conventional images.^[18–20]^ During imaging, medical images are extremely susceptible to interference from external factors, such as noise, organ movement, magnetic field offset, metal artifacts, and differences in tissue structure and location in different human bodies.^[21–27]^ These factors lead to tissues of interest in some images. The contrast decreases, and the edge part has a certain degree of ambiguity and unevenness. Therefore, the precise segmentation of medical images is a very challenging scientific research task.^[28–46]^ The segmentation of medical images generally requires high integrity and accuracy of the results. Usually, after the preliminary imaging is completed, certain technical means are needed to process the results accordingly to show clear and identifiable tissues of interest.

**Figure 3:**
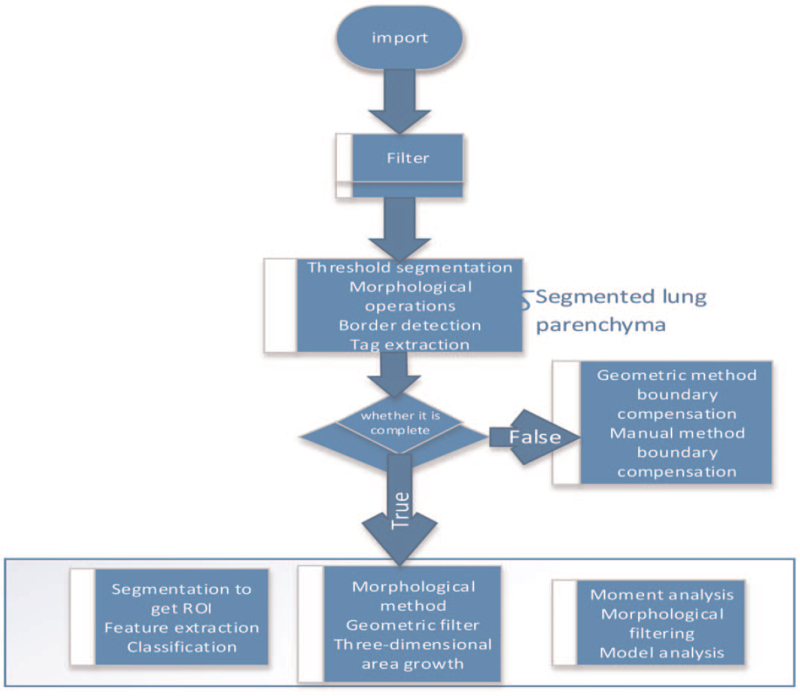
The general processing flow based on the lung medical imaging CAD system.

The CAD system usually includes 4 main stages: lung parenchymal segmentation, candidate lesion area detection, feature extraction and selection, and diagnosis. The classification of the entire system largely depends on the intermediate results of image processing, and the precise segmentation of lung parenchyma is the key to subsequent lung lesion detection and classification. The general processing flow based on the lung medical imaging CAD system is shown in Figure 3.

The methods based on a manual outline by doctors or traditional software outlines are time-consuming and labor-intensive or have poor generalization ability.^[47–49]^ Methods based on deep learning exhibit excellent segmentation performance in medical image segmentation. However, because of medical equipment imaging reasons (imaging artifacts), the structure of the organ lesion itself (body fluid inside the organ lesion, muscle separation, an unclear definition of the edges of adjacent organ lesions), and many other reasons cause the edges of the organ lesions to be segmented to be unclear. Existing deep learning methods have solved these problems. The segmentation methods in COVID-19 applications are mainly divided into 2 categories: methods for lung area and lung disease. The lung area-oriented method aims to separate the lung area (i.e., the entire lung and lung lobes) from other (background) areas in CT or X-rays, which is considered to be the first step in the segmentation of COVID-19 lesions.^[50,51]^ The lung injury-oriented method aims to separate lung lesions (interference from external factors, such as noise, organ movement, magnetic field deviation, and metal artifacts) from the lung area.

### 2.2 Segmentation method

**Figure 4:**
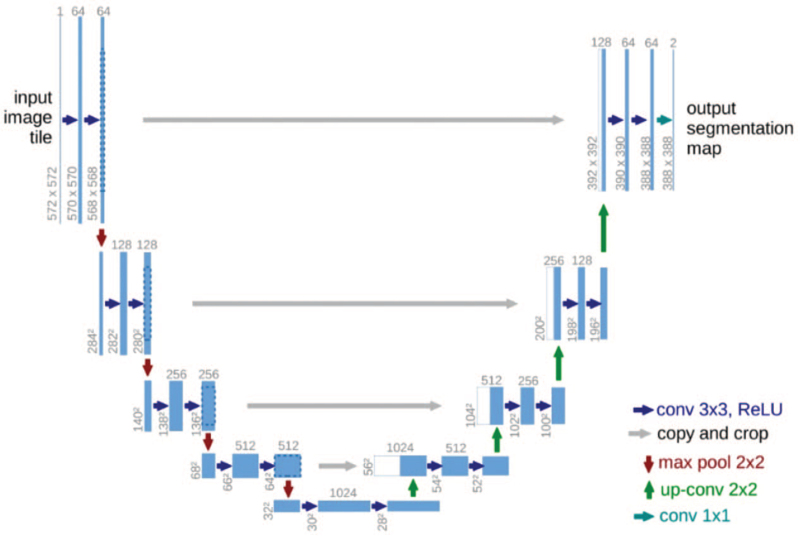
U-Net structure.

Table 2 summarizes the work involving image segmentation in COVID-19 research. U-Net is widely used in medical image segmentation, and many researchers use this network to segment COVID-19 lung images;^[50]^ the network structure is shown in Figure 4. In 2015, Olaf Ronneberger, Philipp Fischer, and Thomas Brox^[63]^ proposed the U-Net network structure and used it for the segmentation of cell images under an electron microscope in the ISBI competition. The generation of U-Net has greatly promoted research on medical image segmentation. U-Net is a fully convolutional network with a U-shaped structure with symmetrical encoding and decoding signal paths. The same layer in the 2 paths is connected by shortcuts. In this case, the network can learn better visual semantics. U-Net uses 4 downsampling (max pooling) and 4 upsampling (transposed convolution) to form a U-shaped SGD+M omentum structure. The loss function is the cross-entropy data preprocessing using mirrored edges, which can be better refined; there is an elastic deformation in the increase of boundary information data, which can deal with small sample datasets for faster and more effective segmentation and can be generalized to many application scenarios. Therefore, it is widely used for the segmentation of COVID-19 lung infection areas. The papers^[50,52–56]^ all used the modification method.

**Table 2:** Application of image segmentation in COVID-19.

Literature	Modality	Method	Target ROI	Application	Highlights
Zheng et al^[50]^	CT	U-Net	Lung	Diagnosis	Weakly-supervised method by pseudo label
Cao et al^[52]^	CT	U-Net	Lung, Lesion	Quantification	Quantitative CT for providing an objective assessment of pulmonary involvement and therapy response in COVID-19
Huang et al^[53]^	CT	U-Net	Lung, Lung lobes, lesion	Quantification	Patients were divided into mild, moderate, severe, and critical types
Qi et al^[54]^	CT	U-Net	Lung lobes, Lesion	Quantification	Intervention CT radiomics models based on logistic regression (LR) and random forest (RF)
Gozes et al^[55]^	CT	U-Net/ Commercial Software	Lung, Leson	Diagnosis	Combination of 2D and 3D methods
Li et al^[56]^	CT	U-Net	Lesion	Diagnosis	differentiate COVID-19 and CAP from chest CT images
Chen et al^[57]^	CT	U-Net++	Lesion	Diagnosis	Greatly improve the efficiency of radiologists in clinical practice
Shuo Jin et al^[58]^	CT	U-Net++	Lung, Lesion	Quantification	Joint segmentation and classification
Shan et al^[59]^	CT	VB-Net	Lung, Lung lobes, Lung segments, Lesion	Quantification	Human-in-the-loop
Tang et al^[60]^	CT	Commercial Software	Lung, Lesion, Trachea, Bronchus	Quantification	The images highlight the distribution of pulmonary lesions
Shen et al^[61]^	CT	Commercial Software	Lesion	Quantification	Quantitative CT analysis for stratifying COVID-19
Tan Y Q^[62]^	CT	Inf-Net	Lung, Lesion	Quantification	Only a small number of labeled images are needed, and unlabeled data is mainly used

Shan et al^[59]^ used VB-Net for segmentation to segment the GGO and real variables. Zhou^[64]^ proposed UNet++, which is much more complicated than U-Net. UNet++ is a new general-purpose image segmentation architecture for more accurate image segmentation.^[65]^ UNet++ consists of U-Nets of varying depths whose decoders are densely connected at the same resolution via the redesigned skip pathways, which aim to address 2 key challenges of the U-Net:

1. unknown depth of the optimal architecture and
2. the unnecessarily restrictive design of skip connections, because the network inserts an embedding between the encoding and decoding path sets of the convolution structure, which can improve the performance of segmentation.

Therefore, training is more difficult and time-consuming. Oktay et al^[66]^ proposed a U-Net that can capture fine structures in medical images, which is suitable for the segmentation of lesions and lung nodules in COVID-19 applications. Training a segmentation network requires sufficient labeled data. In COVID-19 image segmentation, manual labeling of lesions is time-consuming and inefficient; therefore, the segmentation task usually does not have sufficient training data. To solve this problem, a simple method is to combine existing human knowledge for training. For example, Shan et al^[59]^ trained people to use a VB-Net segmentation network. The training of this network involved interactions with radiologists. VB-Net combines the characteristics of the U-Net and the residual network. Smooth gradient flow is easier to optimize and converge. Qi et al^[54]^ used the initial region annotation provided by a radiologist and used U-Net to describe lung lesions. Other studies have used clinical diagnostic knowledge to identify infected areas through attention mechanisms.^[58]^ When the training data are insufficient for segmentation, weakly supervised machine-learning methods are used. For example, Zheng et al^[50]^ suggested the use of unsupervised methods to generate image segmentation masks. Due to the lack of annotations and the widespread existence of medical images in lung segmentation, research on COVID-19 proposes unsupervised and semi-supervised methods to solve the problem of collecting sufficient labeled data for deep model training in a short time.

**Figure 5:**
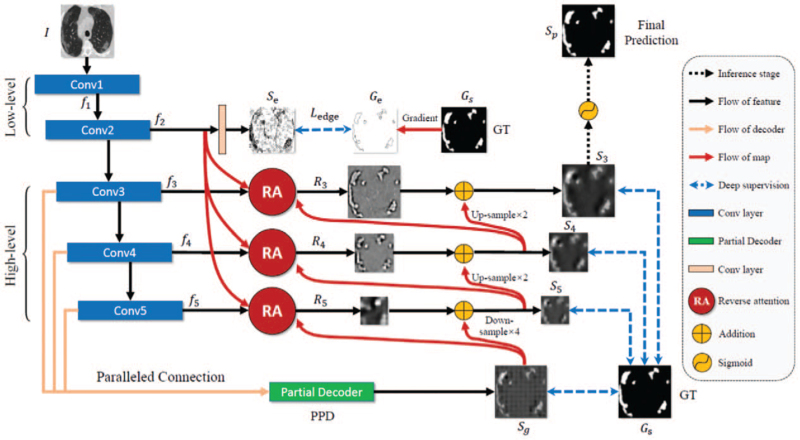
Inf-Net structure.^[62]^

Fan et al^[62]^ proposed a new type of COVID-19 lung infection segmentation deep network (Inf-Net), which can automatically identify the infected area from chest CT slices. In the network, a parallel partial decoder is used to aggregate the high-level features and generate global graphs. Then, implicit inverse attention and explicit edge attention are used to model and enhance the characterization of the boundary. The network structure is illustrated in Figure 5 CT images are first sent to 2 convolutional layers to extract high-resolution, semantically weak (low-level) features. Here, an edge attention module is added to improve the representation of the boundary of the target area. Then, the low-level features obtained by f2 are fed into 3 convolutional layers to extract high-level features. First, the parallel partial decoder (PPD) is used to aggregate these features and generate a global map Sg of the rough location of lung infections. Second, these features combined with f2 are fed to multiple reverse attention (RA) modules under the guidance of Sg. The RA modules are organized in a cascaded manner. For example, R4 depends on the output of another RA (R5). The output of the last RA (s3) is input into the activation function to predict the area of lung infection. Use standard binary cross-entropy to constrain the predicted edge mapping and ground truth (GT) edge mapping:

$$L_{edge}=-\sum_{x=1}^{w}\sum_{y=1}^{h}\left[G_{e}\log(S_{e}\right)+(1-G_{e})\log(1-S_{e})$$

Edge information can provide useful constraints for segmentation to guide feature extraction. The advantage is that considering that the low-level features (in f2) retain sufficient edge information, the low-level feature f2 with medium resolution is provided to the edge attention (EA) module to highlight the learning edge. That is, feature f2 undergoes a convolution layer to generate an edge map.

### 2.3 Application of segmentation in coronavirus disease 2019

**Figure 6:**
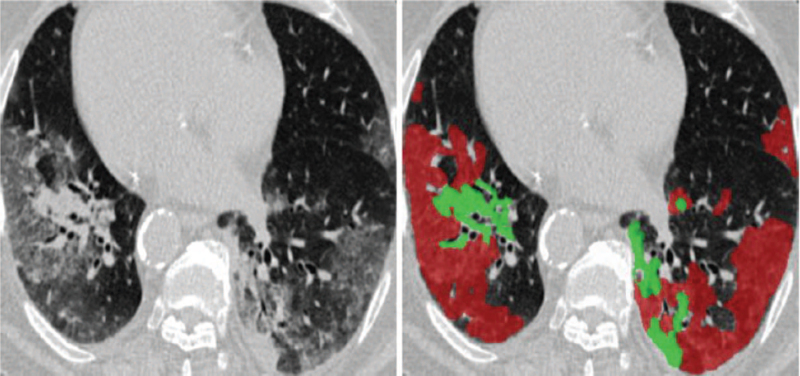
COVID 19-infected region exemplary CT axial slices, wherein the red and green mask and the mask represent consolidation GGO.^[62]^

In the process of lung infection detection, clinicians first roughly locate the infected area and then accurately extract the outline based on the local symptoms. Therefore, the normal area and the junction area are 2 key features that distinguish normal tissue from infection. Various studies have used machine learning-based image segmentation in COVID-19 chest images.^[50,52–59,60,61]^ For example, Li et al used U-Net to segment the lungs and distinguish between COVID-19 and community-acquired pneumonia on chest CT. This is an important auxiliary diagnostic method when medical resources are very tight because of community-acquired pneumonia. Pneumonia and COVID-19 have some common features on chest CT images. Cao et al.^[52]^ used deep learning-based lung segmentation to assess the development of COVID-19. Huang et al. used U-Net to quantitatively evaluate the GGO area to evaluate the infection and the stage,^[53]^ and Qi et al used the U-Net network to assess the stage of the patient's condition and estimate the time required for treatment. The main application of image segmentation is quantization.^[59,60,61]^ Shan et al^[59]^ proposed a VB-Net-based network for the segmentation of lung, lobe, and lung infections. This method can quantify data, including quantitative assessment of follow-up progress, display of the percentage of infection (POI), and three-dimensional visualization of lesion distribution. Tan YQ^[62]^ constructed a COVID-19 semi-supervised infection segmentation (COVID-SemiSeg) dataset, including 100 labeled CT slices from the COVID-19 CT segmentation dataset and 1600 images from the COVID-19 CT acquisition dataset The image is not tagged. A new type of COVID-19 lung CT infection segmentation network, “Ifo-Net,” is proposed, which uses implicit reverse attention and explicit edge attention to improve the identification of infected areas. In addition, a semi-supervised solution, semi-supervised inf-Net, is provided to alleviate the shortage of high-quality labeled data. Extensive experiments on the COVID-SemiSeg dataset and real CT volume show that the performance of the proposed Inf-Net and semi-info-Net is better than that of the cutting-edge segmented transmission model, which improves the latest performance. The system has great potential in COVID-19 diagnosis and evaluation, such as quantifying epidemic areas, monitoring longitudinal disease changes, and large-scale screening and processing. The segmentation effect is illustrated in Figure 6.

In summary, image segmentation based on machine learning is widely used in COVID-19 chest images, which can quantitatively describe the lesion area and infection volume, and can assist doctors in accurately identifying lung infection sites, estimating infection time, and assessing the severity of the infection.

## 3 Application of artificial intelligence in the auxiliary diagnosis

In outbreak areas, patients suspected of COVID-19 urgently need to be isolated for diagnosis and appropriate treatment. Owing to their fast acquisition speed, X-ray and CT scans are widely used to provide an auxiliary diagnosis for radiologists. However, medical images, especially chest CT, contain hundreds of slices, and expert diagnosis takes a long time. Besides, as a new disease, COVID-19 has manifestations similar to those of other types of pneumonia. The texture, size, and location of the infection in CT slices vary greatly, which is challenging for diagnosis, which requires radiologists to accumulate a large amount of experience to achieve a higher diagnostic effect. Therefore, it is necessary to use medical images for artificial intelligence-assisted diagnosis, which can not only alleviate the work pressure of doctors but also help doctors improve the accuracy and efficiency of diagnosis. Table 3 lists the latest relevant research in this area.

**Table 3:** Medical image related research on AI-assisted diagnosis of COVID-19.

	Modality	Subjects	Task	Method	Result
Ghoshal et al^[67]^	X-Ray	70 COVID-19Others (# of subjects notavailable)	Classification: COVID-19/ Others	Bayesian DNN	Accuracy: 92.9%
Narin et al^[68]^	X-Ray	50 COVID-1950 Normal	Classification: COVID-19/Normal	ResNet50, InceptionV3 and Inception- ResNetV2	ResNet50 Accuracy: 98%Inception V3 Accuracy: 97%Inception-ResNetV2 Accuracy::87%
Zhang et al^[69]^	X-Ray	70 COVID-191008 Others931 Bac. Pneu.660 Vir. Pneu.1203 Normal	Classification: COVID-19/Others	ResNet	Sensitivity:96.0%Specificity: 70.7%Accuracy:95.2%
Ezz El-Din Hemdan^[70]^	X-Ray	50 COVID-1925 Normal	Classification: COVID-19/Normal	VGG19/Google MobileNet	Accuracy:91%Sensitivity: 89%
Ozturk T^[71]^	X-Ray	224COVID-19,700 Bac. Pneu.504 Normal	Classification: COVID-19/Bac. Pneu./Normal	DarkNet (YOLO)	Accuracy:98.08%
Chen et al^[57]^	CT	51 COVID-1955 Others	Classification: COVID-19/Others	UNet++	Accuracy: 95.2%Sensitivity:100%
Zheng et al^[50]^	CT	313 COVID-19229 Others	Classification: COVID-19/Others	U-NetDeCoVNet	Sensitivity:90.7%Accuracy: 91.1%Specificity::0.959
Das A K et al^[72]^	CT	496 COVID-191385 Others	Classification: COVID-19/Others	Inceptionv3 architecture	Sensitivity:94.1%Accuracy:95.5%
Jin et al^[58]^	CT	723 COVID-19413 Others	Classification: COVID-19/Others	segmentation-classification	Sensitivity:97.4%Accuracy:92.2%
Wang et al^[73]^	CT	44 COVID-1955 Vir. Pneu.	Classification: COVID-19/Vir. Pneu.	migration-learning	Accuracy: 82.9%
Ying et al^[74]^	CT	88 COVID-19100 Bac. Pneu.86 Normal	Classification: COVID-19/Bac. Pneu./Normal	ResNet-50	Accuracy: 86.0%
Ozcan T. A et al^[75]^	CT	219 COVID-19224 Influ.-A175 Normal	Classification: COVID-19/Influ.-A/Norma	GoogleNet, ResNet18 and ResNet50	Accuracy: 86.7%
Li et al^[56]^	CT	468 COVID-191551 CAP1445 Non-pneu.	Classification: COVID-19/CAP/Non-pneu.	ResNet-50	Sensitivity:90.0%Accuracy:96.0%
Shi et al^[76]^	CT	1658 COVID-191027 CAP	Classification:COVID-19/CAP	CovMUNET	Accuracy: 87.9%Specificity: 90.7%Sensitivity: 83.3%
Chen et al^[77]^	CT	98COVID-19	Classification: COVID-19/Others	Clinical features model, Radiological semantic features model	Accuracy: 94%Specificity: 79%
Wang, Lin, Wong^[78]^	CT	13,645 COVID-19	Classification: COVID-19/Others	COVID-Net: A deep CNN	Accuracy: 92.4%
Xu et al ^[79]^	CT	110COVID-19	Classification: COVID-19/Others	3-dimensional deep learning model	Accuracy: 86.7%
Wang et al^[80]^	CT	1065 CT images (325 COVID-19 and 740 viral pneumonia)	Classification: COVID-19/Others	Modified inception transfer-learning model	Accuracy: 79.3%Specificity: 83%Sensitivity: 67%
Li et al^[77]^	CT	3322 COVID-19	Classification: COVID-19/Others	COV-Net	Accuracy: 95%
Charmaine Butt et^[81]^	CT	528 COVID-19	Classification: COVID-19/Vir. Pneu./Normal	location-attention oriented model	Accuracy: 99.6%Specificity: 92.2%Sensitivity: 98.2%
Zhang et al^[82]^	CT and X-ray	42 COVID-19 patients44 healthy	Classification: COVID-19/Others	end-to-end multiple-input deep convolutional attention network (MIDCAN)	Accuracy: 98.02%Specificity:97.95%Sensitivity: 98.10%
Wang et al^[40]^	CT	284 COVID-19 images,281 community-acquired pneumonia293 secondary pulmonary tuberculosis images;306 healthy images,	Classification: COVID-19/ community-acquired pneumonia/ secondary pulmonary tuberculosis images/ healthy	CCSHNet	Precision: 97.32%Specificity: 95.61%F1 scores:96.46%
Yu et al^[82]^	CT	148 COVID-19148 healthy	Classification: COVID-19/Others	ResGNet	Accuracy: 98.02%Specificity:97.95%Sensitivity: 98.10%

### 3.1 X-ray based Screening of coronavirus disease 2019

Although chest X-rays are not as sensitive as CT images to chest abnormalities, their portability and economic benefits are better than CT imaging, so they are also widely used to study COVID-19 as a way to study patient infection. According to the literature,^[83]^ chest X-rays of COVID-19 pneumonia are different, but they may be bilateral, with lower lobes extending to the surface of the pleura. These functions help distinguish COVID-19 pneumonia from other pathological causes of lung disease. Many researchers have focused on this point and have applied it to machine learning.

Ezz El-Din Hemdan et al^[70]^ improved the visual geometry group network (VGG19) and the second version of Google Mobile Net. Each deep neural network model can analyze the normalized intensity of the X-ray images. The study verified 50 chest X-ray images, of which 25 were confirmed to be positive for COVID-19. To classify the patient's status as COVID-19 negative or positive, the accuracy rate reached 91%. The literature^[71]^ believes that the deep learning model is very sensitive in detecting COVID-19 lung involvement, so the diagnostic accuracy is very high. In the evaluation process using the model, X-ray photographs of COVID-19 patients confirmed to be positive by PCR were used. This model can easily detect GGO, merged areas, and nodular opacity, which are the pathological findings of COVID-19 patients on X-ray photography. In COVID-19, bilateral, lower lobe, and surrounding involvement are observed, and the proposed model can detect the location of the lesion. In addition, it can also evaluate X-rays that are difficult for radiologists to assess with poor quality. Ghoshal et al^[67]^ proposed a Bayesian convolutional neural network to assist in judging uncertainty in the diagnosis of COVID-19. The test results show that the detection accuracy rate of the VGG16 model increased from 85.7% to 92.9% to improve the understanding of the results of deep learning and promote a more accurate decision-making process.

Narin et al^[68]^ used 3 different deep learning models for training, the 3 models are ResNet50, Inception v3, and Inception-ResNetV2. The training set included chest X-ray images of 50 COVID-19 patients and 50 normal chest X-ray images. The training results showed ResNet50 accuracy of 98%, Inception V3 accuracy of 97%, and Inception-ResNetV2 Accuracy: 87%. The ResNet50 model is more accurate than the other 2 networks. Ozturk et al^[71]^ proposed a method for detecting COVID-19 cases based on X-ray images of a deep convolutional neural network (DarkNet). Similarly, from these 2 datasets, the dataset includes chest X-ray images from 504 healthy people, 724 viral pneumonia patients, and 224 COVID-19 patients. The test accuracy of the COVID network is 98.08%.

Most current studies use X-ray images to distinguish COVID-19 from other pneumonia and healthy subjects. Due to the limited number of COVID-19 images, it is not enough to evaluate the robustness of these methods, and it also raises questions about the generalizability of applications in other clinical centers. In addition, the severity of the subjects is difficult to assess; therefore, the work of the app remains on the detection of COVID-19.

### 3.2 Lung computed tomography image screening of coronavirus disease 2019 patients based on machine learning

The main task is: Classification of COVID-19 from non-COVID-19 and other pneumonia

**Figure 7:**
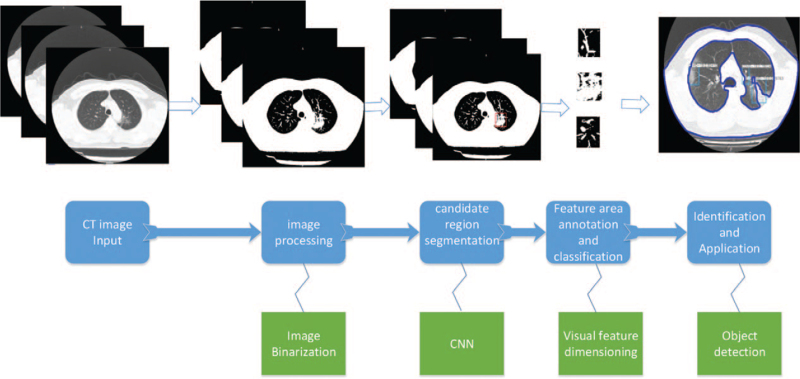
Process flow chart of a deep learning model for classification.

Charmaine et al^[77]^ used a multi-convolutional neural network (CNN) model to classify CT samples with influenza virus COVID-19 and collected the above research and the existing 2D and 3D deep learning models developed, which were compared and combined with the latest clinical understanding; the AUC obtained was 0.996, a sensitivity of 98.2%, and a specificity of 92.2%, and it can distinguish between COVID-19 and common pneumonia. The specific process is illustrated in Figure 7. First, input CT images for preprocessing to extract effective lung regions. The 3D CNN model was then used to train the annotation features. The main data set contained 528 COVID-19 patients, 194 viral pneumonia patients, and 145 healthy individuals. Next, we used the image classification model to classify all images, one of the following 3 types: COVID-19, influenza A virus pneumonia, and no infection. Finally, it is classified according to the weight to obtain the target detection result to realize the recognition and classification of CT images.

Li^[56]^ et al used ResNet50 on 2D slices with shared weights and combined it with max-pooling to discriminate COVID-19 from community-acquired pneumonia (CAP) and non-pneumonia. The data set contained 3322 patients, 1296 of whom had COVID-19, 1735 had community-acquired pneumonia, and 1325 had non-pneumonia, and finally showed a sensitivity of 90%, specificity of 96%, and AUC of 0.96 in identifying COVID-19.

Common pneumonia, especially viral pneumonia and COVID-19, have many similar imaging features, such as GGO and consolidation, which increase the difficulty of machine learning identification. Xu et al^[75]^ used a V-Net-based deep learning model to segment candidate infection areas. The plaques in the infected area were sent to the Resnet-18 network, along with the characteristics of the relative infection distance from the edge. Their study used chest CT images of 219 COVID-19 patients, 224 influenza A patients, and 175 healthy people. The final recognition accuracy was 86.7%., specificity is 67.0%, sensitivity is 74.0%.

Shi et al^[76]^ used VB-Net to segment the image into left and right lungs and manually labeled them to train the random forest model and grouped the detection results based on the size of the infection. The test results showed a sensitivity of 90.7%, specificity of 83.3%, and accuracy of 87.9%.

Zhang et al^[84]^ fusing chest CT with chest X-ray to help improve the AI's diagnosis performance, they created an end-to-end multiple-input deep convolutional attention network (MIDCAN) by using the convolutional block attention module (CBAM), and they have achieved very good results. The sensitivity is 98.10%, the specificity is 97.95%, and the accuracy is 98.02%. Wang et al^[85]^ proposed a novel CCSHNet for COVID-19 detection. Their CCSHNet can achieve the best performance compared to 12 state-of-the-art approaches, and may help radiologists use CCT to diagnose COVID-19 more accurately and faster. Yu et al^[82]^ first attempted at combining graph knowledge into the detection of COVID-19, and their model achieved the best performance compared to SOTA in terms of accuracy. Table 4 shows comparison results of COVID-19 detection and recognition models. The results show that the model MIDCAN of the latest paper^[84]^ achieved the best results in Sensitivity and Accuracy in COVID-19 detection and recognition.

**Table 4:** Comparison results of COVID-19 detection and recognition models.

Method	Specificity	Sensitivity	Accuracy
Modified inception transfer-learning model^[80]^	0.83	0.67	0.793
COV-NET^[77]^	–	–	0.95
STAR methods^[86]^	0.9113	0.9493	0.9249
CovMUNET^[76]^	0.907	0.833	0.879
DeCoVNet^[50]^	0.959	0.911	0.911
COVID-Net^[78]^	–	–	0.924
DeCoVNet^[50]^	**0.979**	0.911	0.959
MIDCAN^[82]^	0.9795	**0.9810**	**0.9802**
ResGNet^[82]^	0.9591	0.9733	0.9662

### 3.3 Assessment of the severity of coronavirus disease 2019

Chest CT images of COVID-19 have been summarized into 4 stages,^[87]^ and 4 evolution stages of chest CT scans have been determined from the onset of symptoms: early (0–4 days) GGO can be observed unilaterally or bilaterally under the pleura at the advanced stage (5–8 days), diffuse GGO, crazy paving, and consolidation can be seen in bilateral multilobes. The peak (9–13 days) dense consolidation was more common and the absorption stage (≥14 days) when the infection was controlled, the absorption period occurred. Consolidation and crazy paving modes are gradually absorbed, leaving only GGO. These imaging findings provide an important basis for evaluating the severity of COVID-19. Severity assessment studies are also important in treatment planning.

Matthew et al^[62]^ analyzed the chest CT images of 176 patients (age, 45.3 ± 16.5 years; 96 males and 80 females) diagnosed with COVID-19, and calculated the total lung infection volume/ratio and 63 quantitative features such as the volume of ground glass shadow (GGO) area. A random forest (RF) model was used to evaluate the severity (non-severe or severe) based on quantitative features. Tang et al^[62]^ and others used machine learning methods to realize the automatic assessment of the severity of COVID-19 (non-severe or severe) based on chest CT images, and explored the relevant features of the severity from the obtained evaluation model. Analyze the chest CT images of 176 patients (age 45.3 ± 16.5 years old, 96 males and 80 females) diagnosed with COVID-19, and calculate the total lung infection volume/ratio, ground glass shadow (GGO) area volume, etc 63 quantitative characteristics. A random forest (RF) model was used to evaluate the severity (non-severe or severe) based on quantitative features. The importance of each quantitative feature reflects the correlation with the severity of COVID-19 and was calculated using the RF model. Through three-time cross-validation, the true positive rate of the RF model was 0.933, the true negative rate was 0.745, the accuracy was 0.875, and the area under the receiver operating characteristic curve (AUC) was 0.91. The importance of quantitative features shows that the volume of ground-glass opacity (GGO) area and its ratio (relative to the entire lung volume) are highly correlated with the severity of COVID-19, and the quantitative features calculated from the right lung were more severe than those from the left lung Degree assessment is relevant. He et al^[40]^ proposed a multi-task and multi-instance learning framework to jointly assess the severity of COVID-19 patients and segment the lung lobes. Their method achieved promising results in severity assessment of COVID-19 patients.

In summary, various studies have been proposed for the diagnosis of COVID-19 based on CT, and the results have been widely used. Screening research for COVID-19 will help in early detection and help reduce the diagnostic uncertainty of radiologists. At the same time, assessing severity helps to arrange treatment plans and plans in advance.

## 4 Public data set

It is worth noting that the number of images used for artificial intelligence algorithm training and testing is still very limited, and the quality of the dataset is insufficient.

The data are the basis of machine learning. Many researchers, medical staff, and patients from all over the world have disclosed COVID-19 information. However, there are not enough X-ray and CT scanning applications for COVID-19, and the data format is not sufficient. Together, these factors limit the application of artificial intelligence to COVID-19. Cohen et al^[88]^ created a dataset containing 123 X-ray COVID-19 cases. In,^[89]^ 288 CT slices were used. The website (http://medicalsegmentation.com/covid19/) contains data sets of 60 patients. One hundred axial CT slices and the magazine “Radiology” (https://pubs.rsna.org/2019-ncov) also published the pictures contained in the papers published in the journal. These datasets are far from sufficient. We hope that more researchers will publish more and better quality datasets, which is conducive to the common fight against COVID-19 by humans, which can promote the application of artificial intelligence algorithm training and testing in COVID-19.

## 5 Discussion

During the global outbreak of COVID-19, many papers and companies have released AI system recognition accuracy data. However, it cannot be said that the 98% accuracy rate of some studies is higher than that of a certain company's 95% because the standards and data sets tested are different.^[90,91,92]^ It is meaningless to directly compare the recognition rate, and CT and X-ray are not absolute standards for judging whether they are infected with COVID-19. In the early days when there were not enough areas to label samples, researchers relied heavily on classification models to identify pneumonia CT. The research conclusion is mainly based on the judgment of the detection and segmentation model, on the one hand, because it is more robust. On the other hand, detecting and segmenting regions can also give doctors better help and assist them in making the most accurate judgments, rather than giving a black box judgment of what percentage of the disease is likely to occur.^[93]^ Among them, the most important thing is to use the category activation map automatically generated during the training of the classification model to locate the most suspicious area and assist the doctor in judgment. The segmentation and detection models have complementary effects. Segmentation of the lesion area can help the detection model to determine the suspected patient, and the suspected patient detected by the classification model can determine the stage of the disease and the severity of the infection through the segmentation model.

## 6 Conclusion

This article outlines the application of AI in X-rays and CT of COVID-19 patients, combined with clinical manifestations and laboratory test results, which will help in the screening, detection, and diagnosis of COVID-19. A medical aid platform based on machine learning can help radiation doctors make clinical decisions and help in screening, diagnosis, and treatment. Through image recognition, segmentation, evaluation, and a combination of clinical manifestations and laboratory test results, it can provide patients with scientific diagnosis and treatment methods. We look forward to more researchers publishing research data and results so that machine learning can play a greater role in the fight against COVID-19.

Currently, the above research has many limitations. There is no uniform standard for detection accuracy, and there is no public training for a large number of datasets. Future research is expected to be conducted. Besides, we expect researchers to diagnose with multimodal medical images and pay attention to the detection of sequelae of COVID-19. According to research,^[94]^ it has been reported that some patients with COVID-19 have heart, lung, and brain sequelae. Therefore, machine learning should also be used to detect and observe sequelae. Getting attention and playing an active role Model promotion U-Net and info-net can also be extended to other related tasks, such as community-based pneumonia segmentation, product defect detection, and pneumonia severity assessment.

## Author contributions

**Conceptualization:** Fengjun Zhang.

**Data curation:** Fengjun Zhang.

**Writing – original draft:** Fengjun Zhang.

**Writing – review & editing:** Fengjun Zhang.
